# Supplementary material for: E3 ligase Nedd4l promotes antiviral innate immunity by catalyzing K29-linked cysteine ubiquitination of TRAF3
Source: Nat Commun. 2021 Feb 19;12:1194. doi: 10.1038/s41467-021-21456-1 (PMC7895832; doi:10.1038/s41467-021-21456-1)
Supplement: Supplementary file 1 — Supplementary information. [file 41467_2021_21456_MOESM1_ESM.pdf]

E3 ligase Nedd4l promotes antiviral innate immunity by catalyzing K29-linked cysteine ubiquitination of TRAF3: Supplementary information

Gao P. et al

Supplementary Figure 1

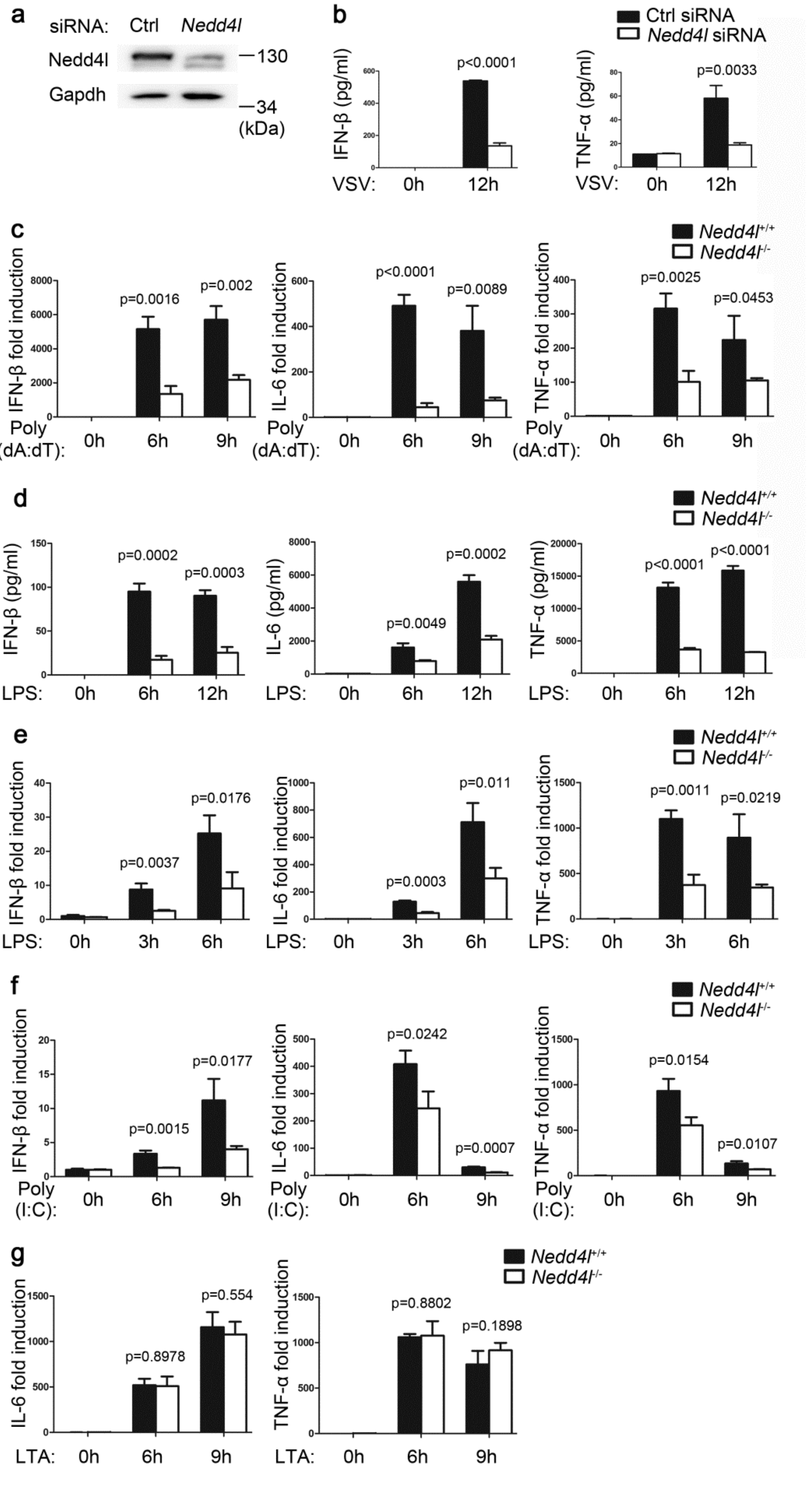

Supplementary Figure 1 Nedd4l deficiency inhibited innate immunity.

(a) IB of Nedd4l or Gapdh in peritoneal macrophages of C57BL/6 transfected with 20 nM control siRNA (Ctrl) or Nedd4l siRNA for 48h.

(b) ELISA assay of IFN-β and TNF-α production in peritoneal macrophages of C57BL/6 transfected with 20 nM control siRNA (Ctrl) or Nedd4l siRNA, and then infected with VSV (MOI=10)for 12 h.

(c) Realtime RT-PCR analysis of IFN-β，IL-6 and TNF-α mRNA expression in wild type (*Nedd4l*$^{+/+}$) and *Nedd4l*$^{-/-}$peritoneal macrophages transfected with poly(dA:dT) (5μg/ml)for 6 and 9 h.

(d) ELISA assay of IFN-β，IL-6 and TNF-α production in wild type (*Nedd4l*$^{+/+}$) and Nedd4l deficient (*Nedd4l*$^{-/-}$)peritoneal macrophages stimulated with LPS (100ng/ml)for 6 and 12 h.

(e) Realtime RT-PCR analysis of IFN-β，IL-6 and TNF-α mRNA expression in wild type (*Nedd4l*$^{+/+}$) and Nedd4l deficient (*Nedd4l*$^{-/-}$)peritoneal macrophages stimulatedwith LPS (100ng/ml)for 3 and 6 h.

(f) Realtime RT-PCR analysis of IFN-β，IL-6 and TNF-α mRNA expression in wild type (*Nedd4l*$^{+/+}$) and Nedd4l deficient (*Nedd4l*$^{-/-}$) peritoneal macrophages stimulated with poly(I:C) (20μg/ml)for 6 and 9 h.

(g) Realtime RT-PCR analysis of IL-6 and TNF-α mRNA expression in wild type (*Nedd4l*$^{+/+}$) and Nedd4l deficient (*Nedd4l*$^{-/-}$) peritoneal macrophages stimulated with LTA (5μg/ml) for 6 and 9 h.

Data are presented as mean ± SD (n=3 per group) and *p*-values by two-tailed unpaired Student’s t-test are indicated in b-g. Results in a-g are representative of three independent experiments.

Supplementary Figure 2

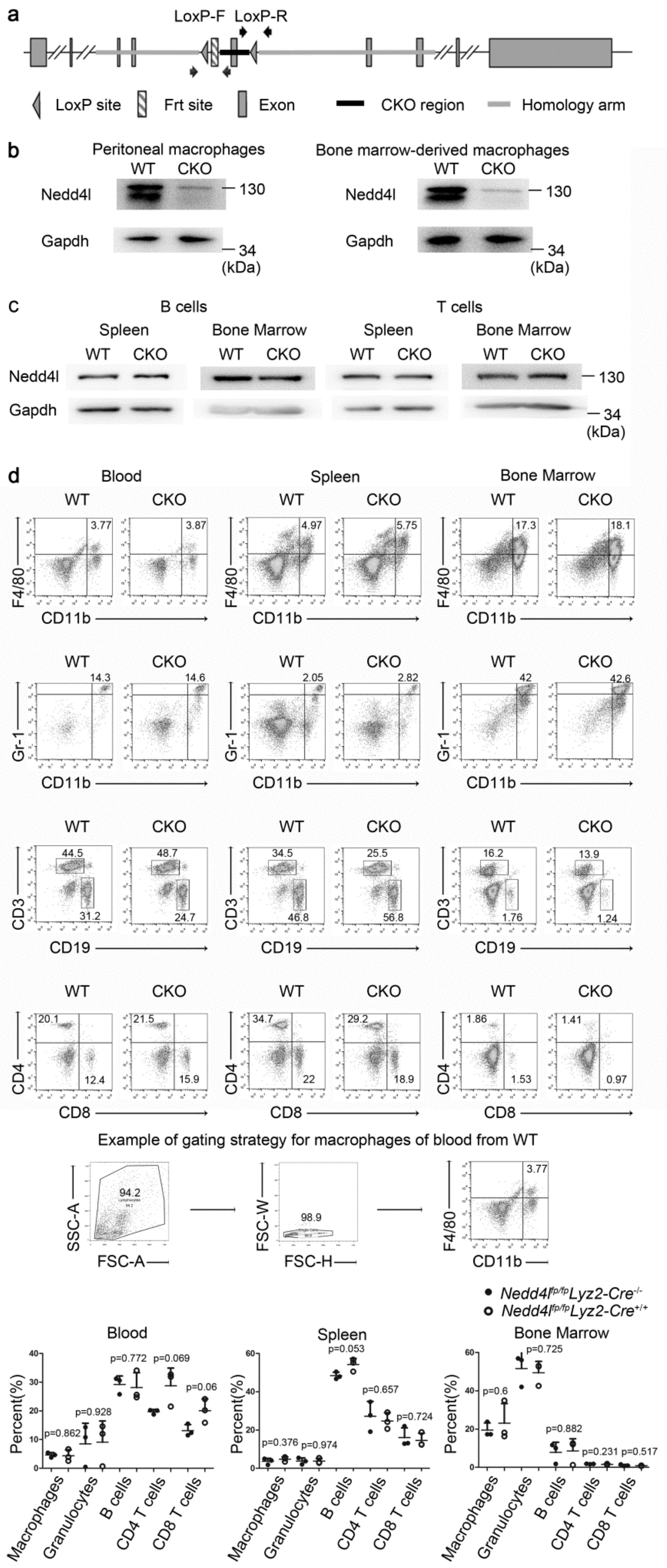

Supplementary Figure 2 Construction and normal immune cells development of Nedd4l conditional knockout mouse.

(a) Gene trap strategy used to construct Nedd4l conditional knockout mouse.

(b) IB analysis of Nedd4l or Gapdh in peritoneal macrophages and BMDM of control *Nedd4l*$^{fp/fp}$*Lyz2-Cre*$^{-/-}$(WT) and *Nedd4l*$^{fp/fp}$*Lyz2-Cre*$^{+/+}$(CKO) mice.

(c) IB analysis of Nedd4l or Gapdh in B cells and T cells from spleen and bone marrow of control *Nedd4l*$^{fp/fp}$*Lyz2-Cre*$^{-/-}$(WT) and *Nedd4l*$^{fp/fp}$*Lyz2-Cre*$^{+/+}$(CKO) mice.

(d) Flow cytometric analysis of the frequency of macrophages (F4/80^{+}CD11b^{+}), granulocytes (Gr-1^{+}CD11b^{+}), B cells (CD19^{+}), CD4 and CD8 T cells (gated on CD3^{+}cells) from spleen, bone marrow and peripheral blood of control *Nedd4l*$^{fp/fp}$*Lyz2-Cre*$^{-/-}$(WT) and *Nedd4l*$^{fp/fp}$*Lyz2-Cre*$^{+/+}$(CKO) mice.

Data are presented as mean ± SD (n=3 per group) and *p*-values by two-tailed unpaired Student’s t-test are indicated in d. Results in b-d are representative of three independent experiments.

Supplementary Figure 3

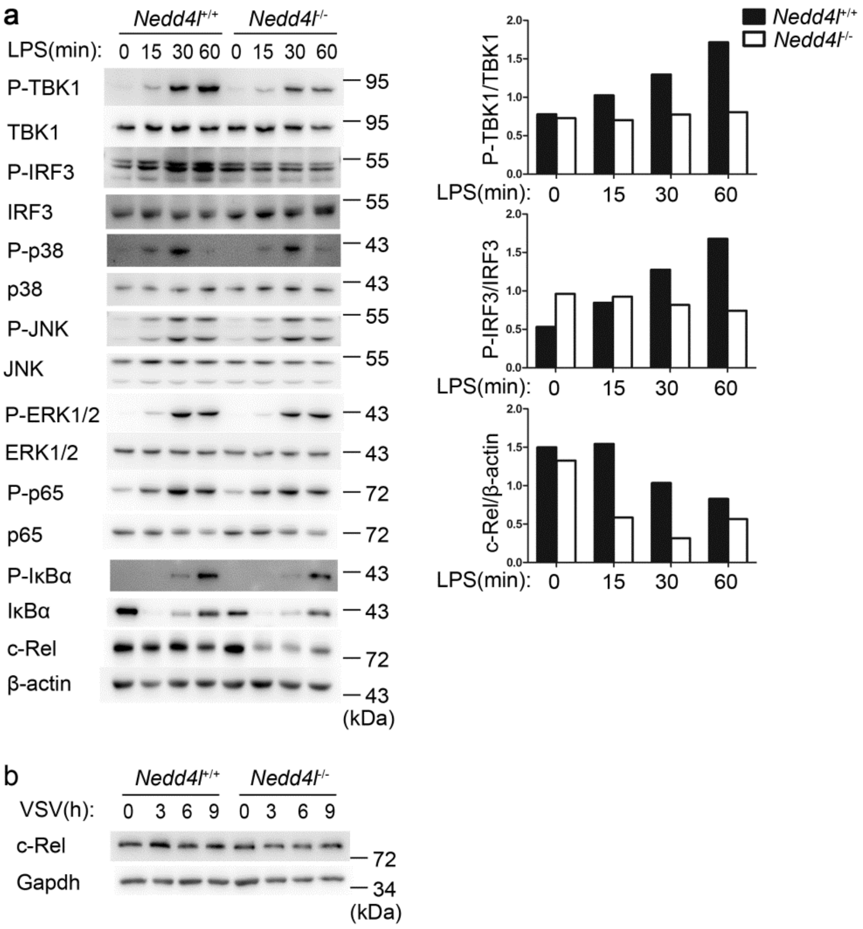

Supplementary Figure3 Nedd4l deficiency inhibited LPS signaling.

(a) Peritoneal macrophages from wild type (*Nedd4l*$^{+/+}$) and Nedd4l deficient (*Nedd4l*$^{-/-}$) mice were stimulated with LPS (100ng/ml) for indicated time. Phosphorylated and total TBK1, IRF3, p38, JNK, ERK1/2, NF-kB p65, IκBα，c-Rel and β-actin were detected by westernblot. Intensities of p-TBK1, TBK1, p-IRF3, IRF3, c-Rel and β-actin signals in the three independent experiments were quantified with Image J and averages of the signals were shown in graphs.

(b) Peritoneal macrophages from wild type (*Nedd4l*$^{+/+}$) and Nedd4l deficient (*Nedd4l*$^{-/-}$) mice were infected with VSV (MOI=10) for indicated time. c-Rel and

Gapdh were detected by westernblot.

Results in a-b are representative of three independent experiments.

Supplementary Figure 4

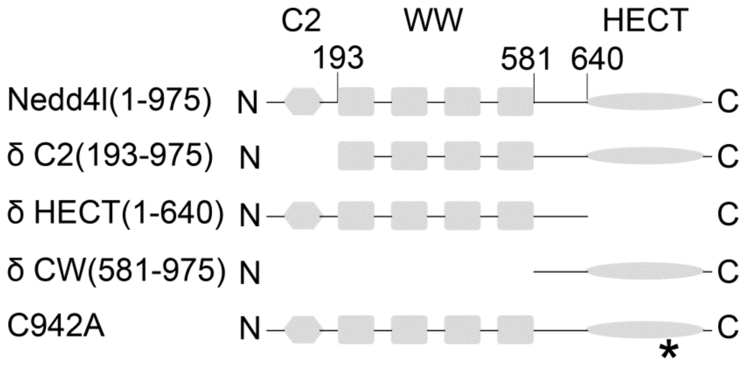

Supplementary Figure 4 Schematic diagram of wild type and mutant Nedd4l.

Nedd4lΔC2 expressed truncated Nedd4l lacking C2 domain. Nedd4lΔHECT lacked C-terminal HECT domain. Nedd4lΔCW lacked C2 domain and the intermediate WW domain. Cysteine 942 in HECT domain of wild type Nedd4l was mutated to alanine residue to construct Nedd4l-C942A. Numbers labeled indicate amino acid residues in the constructs.

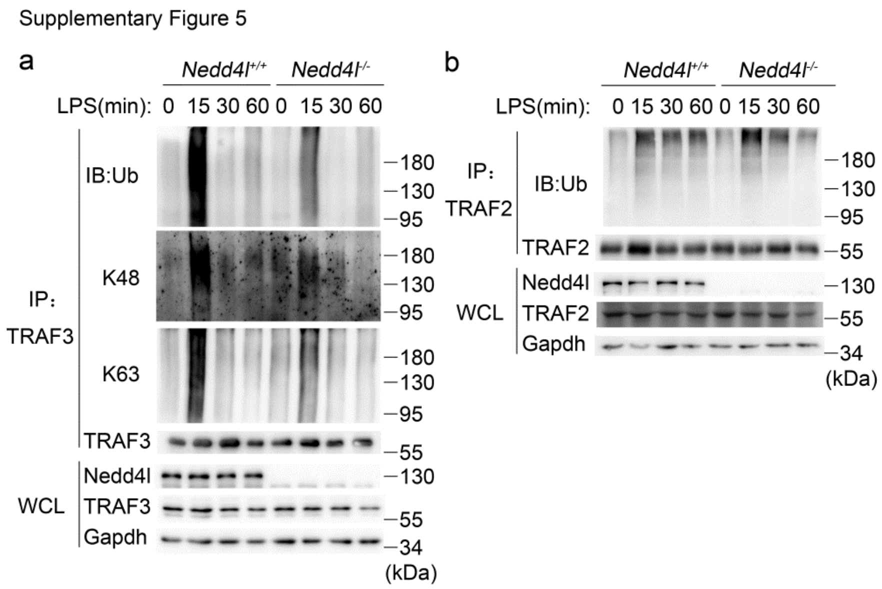

Supplementary Figure 5 Nedd4l deficiency deceased LPS-induced TRAF3ubiquitinationin macrophages.

IB analysis of ubiquitination of TRAF3 (a), TRAF2 (b) in (*Nedd4l*$^{+/+}$) and Nedd4l deficient (*Nedd4l*$^{-/-}$) peritoneal macrophages treated with MG-132 (30μM) and then stimulated with LPS (100ng/ml) for indicated time.

Results in a-b are representative of three independent experiments.

Supplementary Figure 6

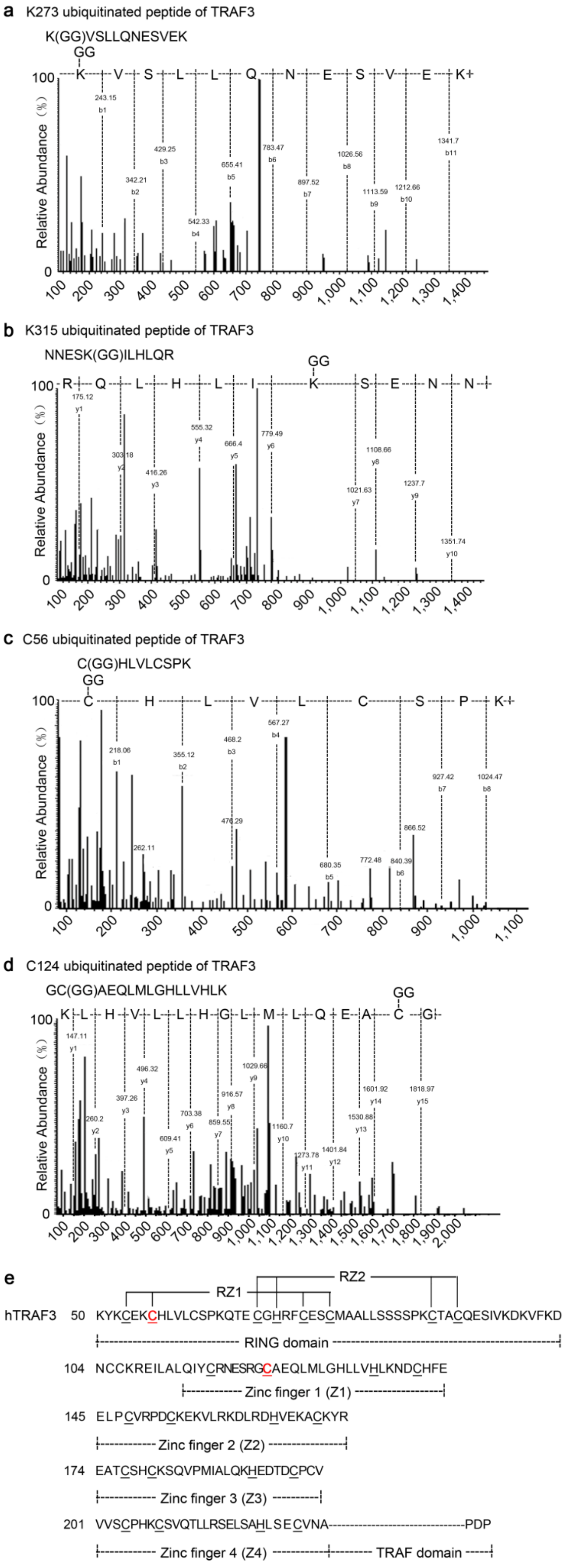

Supplementary Figure 6 Nedd4l-catalysed ubiquitination sites of TRAF3 using LC-MS/MS.

(a,b,c,d) Mass spectrometry analysis of the ubiquitination of TRAF3in 293T cells transfected with TRAF3 with or withoutNedd4l and challenged by VSV(MOI=10); the peptide sequences show the position of candidate ubiquitinated acid residues [K273 (a), K315 (b), C56 (c), C124 (d)].

(e) Zinc fingers inhuman TRAF3 protein. Zinc-coordinating residues in the RING and zinc fingers 1-4 domains are underlined. C56 (red), together with C53, C73 and C76, form zinc finger RZ1. C124 (red), together with C117, H136 and C141, form zinc finger Z1.

Supplementary Figure 7

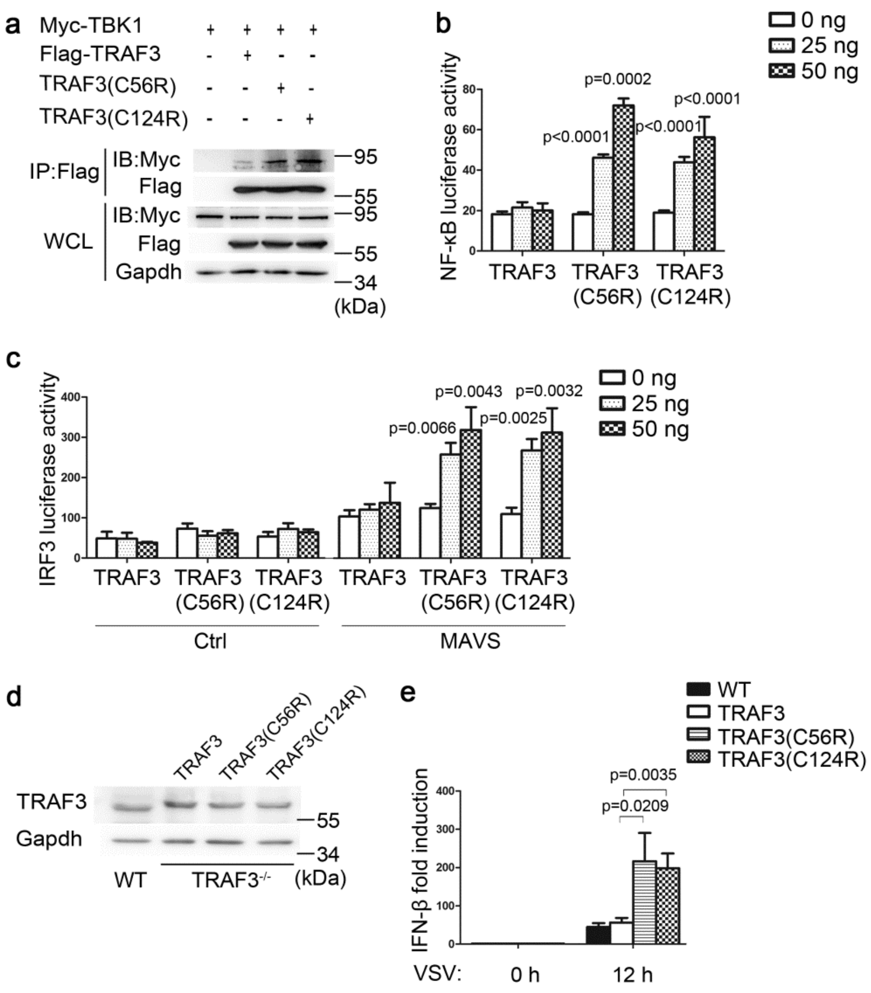

Supplementary Figure 7 Mutation of C56 and C124 enhances TRAF3-mediated signaling.

(a) IB analysis of Myc-tagged TBK1 and Flag-TRAF3 co-immunoprecipitated with Flag-tag specific antibody from lysates of 293T cells co-transfected with plasmids expressing Myc-TBK1 and Flag-tagged wild type TRAF3 or mutant TRAF3 (C56R or C124R).

(b) HEK293 cells were transfected with 100 ng NF-κB luciferase reporter plasmid, 2.5 ng pTK-Renilla-luciferase, together with indicated dose of wild type TRAF3 or mutant TRAF3 (C56Ror C124R)-expressing plasmid. Total amounts of plasmid DNA

were equalized using control empty vector. After 24 h of culture, luciferase activity was measured and normalized by Renilla luciferase activity. Data are shown as mean ±SD (n=4 per group) of one typical experiment.

(c) HEK293 cells were transfected with 50 ng MAVS-expressing plasmid,100 ng IRF3 luciferase reporter plasmid, 2.5 ng pTK-Renilla-luciferase, together with indicated dose of wild type TRAF3 or mutant TRAF3 (C56Ror C124R)-expressing plasmid. Total amounts of plasmid DNA were equalized using control empty vector. After 24 h of culture, luciferase activity was measured and normalized by Renilla luciferase activity. Data are shown as mean ±SD (n=4 per group) of one typical experiment.

(d) IB analysis of TRAF3 in HEK293T cells (WT) and TRAF3-KO HEK293T cells ($TRAF3^{-/-}$) transfected with wild type TRAF3 or mutant TRAF3 (C56Ror C124R)-expressing plasmid.

(e) Realtime RT-PCR analysis of IFN-βmRNA expression in HEK293T cells (WT) and TRAF3-KO HEK293T cells ($TRAF3^{-/-}$) transfected with plasmids expressing TRAF3 or mutants of TRAF3 and then infected with VSV (MOI=10) for 12 h. Data are shown as the mean ± SD (n=3 per group).

Data are presented as mean ± SD in b, c, e and *p*-values by one-way ANOVA followed by Dunnett-t test are indicated in b, c and *p*-value by two-tailed unpaired Student’s t-test are indicated in e. Results in a-e are representative of three independent experiments.
