# Supplementary material for: E3 ligase Nedd4l promotes antiviral innate immunity by catalyzing K29-linked cysteine ubiquitination of TRAF3
Source: Nat Commun. 2021 Feb 19;12:1194. doi: 10.1038/s41467-021-21456-1 (PMC7895832; doi:10.1038/s41467-021-21456-1)
Supplement: Supplementary file 3 — Reporting summary. [file 41467_2021_21456_MOESM3_ESM.pdf]

nature research

Corresponding author(s): Huazhang An, Hongbin Yuan

Last updated by author(s): Nov 2, 2020

# Reporting Summary

Nature Research wishes to improve the reproducibility of the work that we publish. This form provides structure for consistency and transparency in reporting. For further information on Nature Research policies, see Authors & Referees and the Editorial Policy Checklist .

## Statistics

For all statistical analyses, confirm that the following items are present in the figure legend, table legend, main text, or Methods section.

| n/a | Confirmed | |
|---|---|---|
| ☐ | ☒ | The exact sample size (*n*) for each experimental group/condition, given as a discrete number and unit of measurement |
| ☐ | ☒ | A statement on whether measurements were taken from distinct samples or whether the same sample was measured repeatedly |
| ☐ | ☒ | The statistical test(s) used AND whether they are one- or two-sided<br>*Only common tests should be described solely by name; describe more complex techniques in the Methods section.* |
| ☐ | ☒ | A description of all covariates tested |
| ☐ | ☒ | A description of any assumptions or corrections, such as tests of normality and adjustment for multiple comparisons |
| ☐ | ☒ | A full description of the statistical parameters including central tendency (e.g. means) or other basic estimates (e.g. regression coefficient) AND variation (e.g. standard deviation) or associated estimates of uncertainty (e.g. confidence intervals) |
| ☐ | ☒ | For null hypothesis testing, the test statistic (e.g. *F*, *t*, *r*) with confidence intervals, effect sizes, degrees of freedom and *P* value noted<br>*Give P values as exact values whenever suitable.* |
| ☒ | ☐ | For Bayesian analysis, information on the choice of priors and Markov chain Monte Carlo settings |
| ☒ | ☐ | For hierarchical and complex designs, identification of the appropriate level for tests and full reporting of outcomes |
| ☒ | ☐ | Estimates of effect sizes (e.g. Cohen's *d*, Pearson's *r*), indicating how they were calculated |

*Our web collection on statistics for biologists contains articles on many of the points above.*

## Software and code

Policy information about availability of computer code

| | |
|---|---|
| Data collection | No software was used. |
| Data analysis | Software of GraphPad Prism 8(version 8.0.1, GraphPad Software Inc., San Diego, CA, USA), FlowJo(version 10.0.7, Tree Star Inc.,USA) , Image J(version 1.4.3.67, Rawak Software Inc., Stuttgart, Germany) and Adobe Illustrator CC 2017(Adobe Inc.,USA) were used. |

For manuscripts utilizing custom algorithms or software that are central to the research but not yet described in published literature, software must be made available to editors/reviewers. We strongly encourage code deposition in a community repository (e.g. GitHub). See the Nature Research guidelines for submitting code & software for further information.

## Data

Policy information about availability of data

All manuscripts must include a data availability statement. This statement should provide the following information, where applicable:

- Accession codes, unique identifiers, or web links for publicly available datasets
- A list of figures that have associated raw data
- A description of any restrictions on data availability

All data supporting the findings of this study are available within the paper and its supplementary information files.
The source data underlying Figs 1, 2, 3, 4, 5, 6, 7 and Supplementary Figs 1, 2b-d, 3, 5, 6a-d, 7 are provided as a Source Data file.

# Field-specific reporting

Please select the one below that is the best fit for your research. If you are not sure, read the appropriate sections before making your selection.

# Life sciences study design

All studies must disclose on these points even when the disclosure is negative.

| | |
|---|---|
| Sample size | No statistical methods were used to predetermined sample size. Sample size was chosen by following the literature in the field. |
| Data exclusions | No data were excluded from the analyses. |
| Replication | All attempts at replication were successful. At least three biologically independent samples in each group were tested in all experiments except LC-MS/MS in Supplementary figure 6. All independent scores indicated in the figures are biologically independent.. |
| Randomization | We splitted mice equally and randomly allocated to each of experimental group in vivo according to their genotype. Randomization was not relevant for other experiments, as they were performed in cell lines. |
| Blinding | The investigators were blinded to group allocation during data collection or analysis. |

# Reporting for specific materials, systems and methods

We require information from authors about some types of materials, experimental systems and methods used in many studies. Here, indicate whether each material, system or method listed is relevant to your study. If you are not sure if a list item applies to your research, read the appropriate section before selecting a response.

## Materials & experimental systems

| n/a | Involved in the study | |
|---|---|---|
| ☐ | ☒ | Antibodies |
| ☐ | ☒ | Eukaryotic cell lines |
| ☒ | ☐ | Palaeontology |
| ☐ | ☒ | Animals and other organisms |
| ☒ | ☐ | Human research participants |
| ☒ | ☐ | Clinical data |

## Methods

| n/a | Involved in the study | |
|---|---|---|
| ☒ | ☐ | ChIP-seq |
| ☐ | ☒ | Flow cytometry |
| ☒ | ☐ | MRI-based neuroimaging |

# Antibodies

Antibodies used

P-TBK1 (Ser172) antibody: Cell Signaling Technology, #5483, Rabbit, 1:1000, Western blot;
P-IRF3 (Ser396) antibody: Cell Signaling Technology, #4947, Rabbit, 1:1000, Western blot;
P-ERK1/2 (Thr202/Tyr204,) antibody: Cell Signaling Technology, #9101, Rabbit, 1:1000, Western blot;
P-p38 (Thr180/Tyr182) antibody: Cell Signaling Technology, #9211, Rabbit, 1:1000, Western blot;
P-p65 (Ser536) antibody: Cell Signaling Technology, #3033, Rabbit, 1:1000, Western blot;
P-JNK (Thr183/Tyr185) antibody: Cell Signaling Technology, #9251, Rabbit, 1:1000, Western blot;
P-IkappaBa(Ser32) antibody: Cell Signaling Technology, #2859, Rabbit, 1:1000, Western blot;
TBK1 antibody: Cell Signaling Technology, #3504, Rabbit, 1:1000, Western blot;
IRF3 antibody: Cell Signaling Technology, #4302, Rabbit, 1:1000, Western blot;
JNK antibody: Cell Signaling Technology, #9252, Rabbit, 1:1000, Western blot;
p65 antibody: Cell Signaling Technology, #8242, Rabbit, 1:1000, Western blot;
IkappaBa antibody: Cell Signaling Technology, #4814, Rabbit, 1:1000, Western blot;
Nedd4l antibody: Cell Signaling Technology, #4013, Rabbit, 1:1000, Western blot;
TRAF3 antibody: Cell Signaling Technology, #4729, Rabbit, 1:1000, Western blot and IP;
c-Rel antibody: Cell Signaling Technology, #12707, Rabbit, 1:1000, Western blot;
Ubiquitin antibody: Cell Signaling Technology, #3936, Rabbit, 1:1000, Western blot;
K63-linkage specific polyubiquitin antibody: Cell Signaling Technology, #5621, Rabbit, 1:1000, Western blot;
K48-linkage specific polyubiquitin antibody: Cell Signaling Technology, #4289, Rabbit, 1:1000, Western blot;
Myc antibody: Cell Signaling Technology, #71D10, Rabbit, 1:1000, Western blot and IP;
β-actin antibody: Santa Cruz Biotechnology, #sc-1616, Goat, 1:5000, Western blot;
normal rabbit IgG antibody: Santa Cruz Biotechnology, #sc-2027, 1:1000, IP;
p38 antibody: Santa Cruz Biotechnology, #sc-535, Rabbit, 1:1000, Western blot;
TRAF2 antibody: Santa Cruz Biotechnology, #sc-877, Rabbit, 1:1000, Western blot and IP;
cIAP1/2 antibody: Santa Cruz Biotechnology, #sc-12410, Rabbit, 1:1000, Western blot;
ERK1/2 antibody: Santa Cruz Biotechnology, #sc-93, Rabbit, 1:1000, Western blot;
Flag Tag antibody: Sigma, #F1804, Mouse, 1:10000, Western blot and IP;
HA Tag (26183) antibody: Sigma, #26183, Mouse, 1:10000, Western blot and IP;
Anti-Gapdh antibody: Proteintech, #6004, Mouse, 1:20000, Western blot;
HECTD3 antibody: Proteintech, #11487-1-AP, Rabbit, 1:1000, Western blot;

CD4-PE antibody: BioLegend, #100408, 1:100, Flow cytometry;
CD19-APC antibody: BioLegend, #302211, 1:100, Flow cytometry;
CD11b-APC antibody: BioLegend, #101212, 1:100, Flow cytometry;
CD8-FITC antibody: eBioscience, #11-0081-82, 1:100, Flow cytometry;
Ly-6G(Gr-1)-PE antibody: eBioscience, #12-5931-82, 1:100, Flow cytometry;
F4/80-APC antibody: eBioscience, #17-4801-82, 1:100, Flow cytometry;
CD11b-FITC antibody: eBioscience, #11-0112-81, 1:100, Flow cytometry;
CD3-FITC antibody: BD, #561827, 1:100, Flow cytometry;

Validation

All antibodies have been validated by the manufacturer. We only used antibodies recommended by the manufacturer for the species and application mentioned above.

# Eukaryotic cell lines

Policy information about cell lines

Cell line source(s)

HEK293 cells (ATCC: CRL-1573) and HEK293T cells (ATCC: RCB-2202) were provided by Shanghai Zhong Qiao Xin Zhou Biotechnology Corp., Ltd., and originally purchased from American Type Culture Collection (ATCC).

Authentication

Certificate of STR Analysis was provided by Shanghai Zhong Qiao Xin Zhou Biotechnology Corp., Ltd.

Mycoplasma contamination

All cell lines tested negative for mycoplasma contamination.

Commonly misidentified lines
(See ICLAC register)

No commonly misidentified cell lines are used in the study.

# Animals and other organisms

Policy information about studies involving animals; ARRIVE guidelines recommended for reporting animal research

Laboratory animals

C57BL/6 mice: female, 6-8 weeks old; Nedd4l-deficient mice and littermate control mice: Balb/c, male or female, 6-8 weeks old.Nedd4l conditional knockout mice and littermate control mice: C57BL/6, male or female, 6-8 weeks old. All mice were bred and maintained a room temperature of 20~25ڂ degree Celsius, a relative humidity of 60%, and a 12 hours dark/12 hours light cycle.

Wild animals

The study did not involve wild animals.

Field-collected samples

The study did not involve samples collected from the field.

Ethics oversight

All studies were conducted in accordance with the National Institute of Health Guide for the Care and Use of Laboratory Animals with approval of the Scientific Investigation Board of Second Military Medical University, Shanghai.

Note that full information on the approval of the study protocol must also be provided in the manuscript.

# Flow Cytometry

## Plots

Confirm that:

- [x] The axis labels state the marker and fluorochrome used (e.g. CD4-FITC).
- [x] The axis scales are clearly visible. Include numbers along axes only for bottom left plot of group (a 'group' is an analysis of identical markers).
- [x] All plots are contour plots with outliers or pseudocolor plots.
- [x] A numerical value for number of cells or percentage (with statistics) is provided.

## Methodology

Sample preparation

Single-cell suspensions of macrophages, granulocytes, B cells and T cells were prepared from spleen, bone marrow and peripheral blood and then stained using a subset of antibodies.

Instrument

Cells were analyzed on a Attune NxT (Invitrogen).

Software

The data was analysed using FlowJo software(version 10.0.7, Tree Star Inc.,USA).

Cell population abundance

The abundance of the relevant cell population is obvious, so the purity of the samples was not included.

Gating strategy

The preliminary FSC-A/SSC-A gates were used for the starting cell population and FSC-H/FSC-W gates were used for removing adhesion. Then macrophages were stained with F4/80-APC and CD11b-FITC. Granulocytes were stained with Gr-1-PE and CD11b-APC. T cells were stained with CD3-FITC, CD4-PE and CD8-FITC. B cells were stained with CD19-APC.

☒ Tick this box to confirm that a figure exemplifying the gating strategy is provided in the Supplementary Information.
